# Determination of the erythrocyte sedimentation rate using the hematocrit‐corrected aggregation index and mean corpuscular volume

**DOI:** 10.1002/jcla.24877

**Published:** 2023-04-01

**Authors:** Makoto Higuchi, Nobuo Watanabe

**Affiliations:** ^1^ Functional Control Systems Course, Graduate School of Engineering and Science Shibaura Institute of Technology Saitama Japan; ^2^ Ogino Memorial Laboratory Nihon Kohden Corporation Saitama Japan

**Keywords:** erythrocyte aggregation, erythrocyte sedimentation rate, hematocrit correction, mean corpuscular volume, syllectometry

## Abstract

**Background:**

Determination of the erythrocyte sedimentation rate (ESR) by measurement of erythrocyte aggregation is an alternative to the Westergren method and can be performed rapidly. However, its principle is opaque and the ESR values obtained can deviate from Westergren method values (WG ESR) due to hematocrit. Furthermore, WG ESR is affected by particle size, but no studies have examined the effect of individual mean corpuscular volumes (MCVs).

**Methods:**

Simultaneous measurement of the erythrocyte aggregation index (AI) over a 5‐s interval and determination of the complete blood count in 80 μL blood from 203 patients were performed (hematocrit, 21.4%–52.3%; MCV, 62.7–114.1 fL). ESR values were calculated with the hematocrit‐corrected AI (HAI) for comparison with WG ESR. We improved the calculation formula by using MCV.

**Results:**

The sedimentation velocity of a single erythrocyte in the samples agreed well with an exponential function of HAI. ESR values calculated using HAI showed excellent correlation with WG ESR (*r* = 0.899, *p* < 0.001; Bland–Altman analysis: bias 2.76, limits of agreement (LOA) −24.5 to 30.0), but the difference between the calculated ESR and WG ESR increased with decreasing MCV. Calculation of ESR considering both HAI and MCV eliminated the MCV‐dependent deviation and improved the correlation with WG ESR (*r* = 0.920, *p* < 0.001, bias −2.17, LOA −24.6 to 20.3).

**Conclusion:**

Calculation using HAI and MCV can rapidly provide ESR values that are highly correlated with WG ESR in clinical specimens over a wide range of hematocrit and MCV values.

## Graphical abstract

To determine the erythrocyte sedimentation rate (ESR), simultaneous measurement of the aggregation index (AI) over a 5‐s interval and determination of the complete blood count in 80 μL of blood from 203 patients were performed. Calculation using the hematocrit‐corrected AI and mean corpuscular volume demonstrated that the ESR was highly correlated with the Westergren method in a short period of time (*r* = 0.920). Our results can improve the throughput and efficiency of the clinical workflow and reduce blood collection volumes.

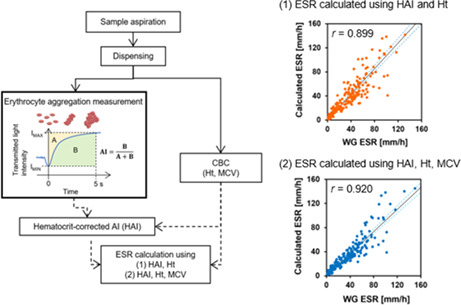

## 1 INTRODUCTION

The erythrocyte sedimentation rate (ESR) is a hematological test for measuring inflammatory activity in the body.^1^ The sedimentation curve is sigmoidal and comprises three phases: the lag phase, sedimentation phase, and packing phase.^2^ Under low shear flow, erythrocytes dispersed in plasma form rouleaux through interactions with inflammatory proteins such as fibrinogen and immunoglobulins and grow as aggregates over time, increasing the sedimentation rate.^3^

Because the Westergren method, the international reference method for ESR measurement,^4^ is manual and has a long test duration of 1 h, various methods and automated analyzers have been developed to shorten the measurement time and improve usability.^5^, ^6^, ^7^, ^8^, ^9^ In particular, a method for the rapid optical measurement of erythrocyte aggregation, called syllectometry or capillary photometry, can estimate ESR in a very short time.^10^, ^11^ Syllectometry provides aggregation parameters calculated from a syllectogram, which is the transmitted or reflected light‐intensity waveform caused by the formation of erythrocyte aggregates.^12^ However, ESR values obtained from this rapid measurement method have been reported to deviate from the ESR obtained by the Westergren method (WG ESR) due to the hematocrit (Ht),^13^, ^14^ and the International Council for Standardization in Hematology (ICSH) recommends that attention be paid to the differences in the methods.^15^

One of the reasons for the discrepancy is that the erythrocyte aggregation parameters are Ht dependent^16^, ^17^ and analysis is time dependent.^6^, ^18^ The other is that ESR is Ht dependently decreased due to the effect of hindered settling.^19^, ^20^ In our previous report,^18^ we investigated the effect of Ht and analysis time on an aggregation parameter, namely the aggregation index (AI) determined from the syllectogram, and demonstrated that the effect of Ht on the AI measured over a 5‐s interval could be corrected as the Ht‐corrected AI (HAI) for samples with added fibrinogen. The sedimentation velocity obtained by eliminating the effect of hindered settling could be expressed by the exponential function of the HAI obtained from the 5‐s syllectogram. Moreover, an accurate sedimentation curve could be obtained by calculation based on the modified Stokes' law and HAI.^18^ However, that study used a limited number of fibrinogen concentration‐ and Ht‐adjusted blood samples from healthy volunteers, and the method has not been validated for clinical samples with various plasma proteins and varying mean corpuscular volumes (MCVs). Although ESR increases with an increasing MCV at fixed conditions of Ht and plasma,^21^ no study has quantitatively evaluated the impact of MCV on the sedimentation rate equation for a variety of Ht values.

Even though it was clear that MCV influenced the effective diameter of the aggregates, which determines the sedimentation rate, it was difficult to prove the validity of the use of individual MCVs for ESR calculations without Ht correction because the influence of Ht is greater than that of MCV. In this study, we compared the ESR obtained by the previously developed calculation method using HAI with WG ESR, using clinical specimens to validate our method. Furthermore, we attempted to improve the accuracy of the ESR calculation by using the effective diameter obtained from individual MCVs.

## 2 MATERIALS AND METHODS

### 2.1 Sedimentation theory

The sedimentation velocity of a single particle *V*_s_ is given by Stokes' Equation (1) and increases with the square of the particle diameter.^22^

(1) $$V_{s}=\frac{2\left(\rho_{e}-\rho_{p}\right)g}{9\mu_{p}}R_{\mathrm{ef}}^{2}$$

Here, *ρ*_e_ is the erythrocyte density [kg/m^3^], *ρ*_p_ is the plasma density [kg/m^3^], *g* is the gravitational acceleration [m/s^2^], *R*_ef_ is the effective erythrocyte radius [m], and *μ*_p_ is the plasma viscosity [Pa·s]. In blood, a large number of erythrocytes causes the hindered settling effect and slows down their sedimentation velocity.^23^ The hindered settling coefficient *φ* is defined by Equation (2) using *V*_s_ and the decelerated sedimentation velocity *V*_e_.

(2) $$\begin{array}{c} \varphi \left(\mathrm{Ht}\right)=\frac{V_{e}}{V_{s}} \end{array}$$

In Equation (3), Oka describes the sedimentation velocity at time *t* with the effective radius increasing due to erythrocyte aggregation.^20^ Here, *α* is the dimensionless size parameter of erythrocyte aggregation and *λ* is the time constant [s].

(3) $$\begin{array}{c} V_{e}\left(t\right)=\frac{2\left(\rho_{e}-\rho_{p}\right)g}{9\mu_{p}}\varphi \left(\mathrm{Ht}\right){\left[R_{\mathrm{ef}}\left\{1+\alpha \left(1-e^{-\frac{t}{\lambda }}\right)\right\}\right]}^{2} \end{array}$$

The increase in erythrocyte aggregation converges with time and, after some time, the sedimentation velocity becomes constant, as shown in Equation (4).

(4) $$\begin{array}{c} V_{e}=\frac{2\left(\rho_{e}-\rho_{p}\right)g}{9\mu_{p}}\varphi \left(\mathrm{Ht}\right){\left[R_{\mathrm{ef}}\left(1+\alpha \right)\right]}^{2} \end{array}$$

A hindered settling coefficient was reported by Richardson and Zaki, shown in Equation (5).^24^ For low Reynolds number conditions, such as ESR, *n* = 4.65.

(5) $$\begin{array}{c} \varphi \left(\mathrm{Ht}\right)={\left(1-\mathrm{Ht}\right)}^{n} \end{array}$$

The Ht‐corrected HAI of the erythrocyte aggregation parameter AI obtained from the 5‐s syllectogram measured by our instrument was defined by Equation (6).^18^

(6) $$\begin{array}{c} \mathrm{HAI}=\mathrm{AI}-k\left(\mathrm{Ht}-0.40\right) \end{array}$$

We reported that *V*_s_, obtained by dividing the observed *V*_e_ by the hindered settling coefficient, agrees well with the value expressed in the following regression equation (Equation (7)) using HAI.

(7) $$\begin{array}{c} V_{s}=a{\left({\mathrm{HAI}}_{5}-b\right)}^{4}+c \end{array}$$

Here, *a* and *b* are coefficients and *c* is the settling velocity in the absence of aggregation, that is, *V*_s_. *V*_e_ was obtained from a previously reported empirical formula (*V*_e_ = 0.778 × WG ESR/3600/1000 [m/s]).^18^ The coefficients *a* and *b* were determined by data fitting using *V*_e_ and HAI obtained from 203 samples (*a* = 0.0102, *b* = 0.380).

As previously reported, the settling distance in the packing phase after a constant sedimentation rate was obtained using Mayer's equation. Details of the ESR calculations are as reported in our previous study.^18^

### 2.2 Correction of the sedimentation rate using MCV

To improve the accuracy of ESR estimation, we attempted to determine *R*_ef_ from the MCV of individuals. Because erythrocytes have a biconcave shape, MCV can be described by the long‐axis radius *R*_L_, as shown in Equation (8).

(8) $$\begin{array}{c} \mathrm{MCV}=\frac{4\pi \beta }{3}{R_{L}}^{3} \end{array}$$

Here, *β* is the ratio of the volume of biconcave erythrocytes to the sphere, which was set to 0.295 based on the volume and long‐axis radius of the erythrocyte model reported previously.^25^ Oka reported that the effective radius of a biconcave shape was equal to 0.71*R*_L_.^15^ Therefore, an effective radius from the MCV (*R*_MCV_) is given by Equation (9).

(9) $$\begin{array}{c} R_{\mathrm{MCV}}=0.71\times \sqrt[{3}]{\frac{3\mathrm{MCV}}{4\pi \beta }} \end{array}$$

The modified velocity *V*_m_ using *R*_MCV_ is as in Equation (10).

(10) $$\begin{array}{c} V_{m}=V_{e}{\left(\frac{R_{\mathrm{MCV}}}{R_{\mathrm{ef}}}\right)}^{2} \end{array}$$

### 2.3 Clinical data collection

Clinical data collection was performed at Juntendo University Hospital in accordance with the Declaration of Helsinki and with the approval of the Ethics Review Board (approval no. 17‐085). After medical treatment, patients' residual blood in K2‐EDTA tubes was used with an opt‐out method. A total of 203 blood samples were used for data analysis. Their WG ESR values ranged from 2 to 120 mm, which was required to test the new technology.^26^

### 2.4 Erythrocyte aggregation and complete blood count measurements

**FIGURE 1:**
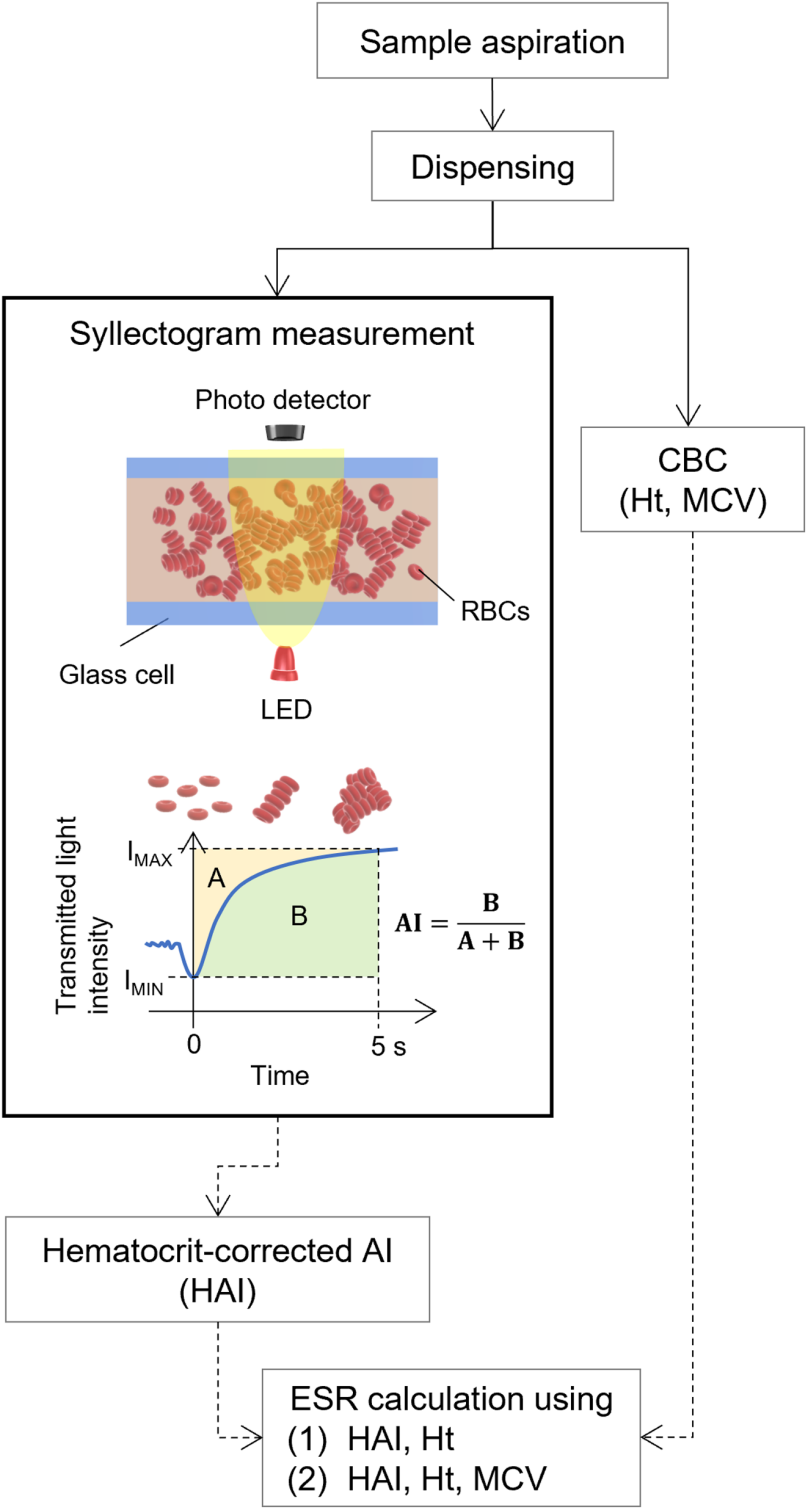
Flowchart of the ESR measurement through a syllectogram and CBC measurement.

A flowchart illustrating the process before the acquisition of ESR values is shown in Figure 1. The analyzer (MEK‐1305; Nihon Kohden Corporation) aspirated 80 μL blood and dispensed 60 μL of the sample into the reservoir connected to the syllectogram measuring unit. The blood sample was withdrawn into a glass cell for optical measurement, and the syllectogram was analyzed to obtain AI, as in the previous report.^18^

At the same time, the total blood count, including Ht and MCV (= Ht [%]/red blood cell count [10^6^/μL]), was measured using the remaining 20‐μL sample through the built‐in complete blood count (CBC) unit, which was similar to the performance‐evaluated product.^27^ All measurements from sample aspiration were performed in duplicate. From the AI and Ht obtained by the measurements, the ESR value was calculated as described in the previous section, and ESR was further corrected by MCV. About 3 min was required to complete the syllectogram and CBC measurements and to rinse the flow path after the measurements were completed.

### 2.5 Determination of ESR by the Westergren method

Reference ESR values were obtained by the Westergren method in accordance with the ICSH recommendations and the Clinical and Laboratory Standards Institute guideline.^15^, ^28^ After the syllectogram and CBC measurement using a portion of the EDTA blood sample, the residual sample was mixed with a 3.2% sodium citrate solution in a 4:1 ratio and was aspirated into a vertically placed plastic Westergren tube (full‐scale length, 200 mm; inner diameter, 2.55 mm; Terumo Corporation). ESR was determined visually as the distance from the top edge to the blood cell layer. The room temperatures were stable during the measurement period within ±1°C. However, because the obtained ESR values were slightly affected by the room temperature, depending on the date (18–25°C), they were corrected to the values measured at 18°C using the environmental temperature correction method.^29^ The samples were measured within 4 h after blood sampling.

### 2.6 Data analysis

Calculation of Pearson's correlation coefficient and data fitting were performed using Microsoft Excel (Microsoft Corporation). Passing–Bablok linear regression analysis and Bland–Altman analysis were performed using XLSTAT (Addinsoft). Student's *t*‐test was used to compare the means between two groups. All *p* values <0.05 were considered statistically significant.

## 3 RESULTS

### 3.1 Relationship between AI and sedimentation velocities

**FIGURE 2:**
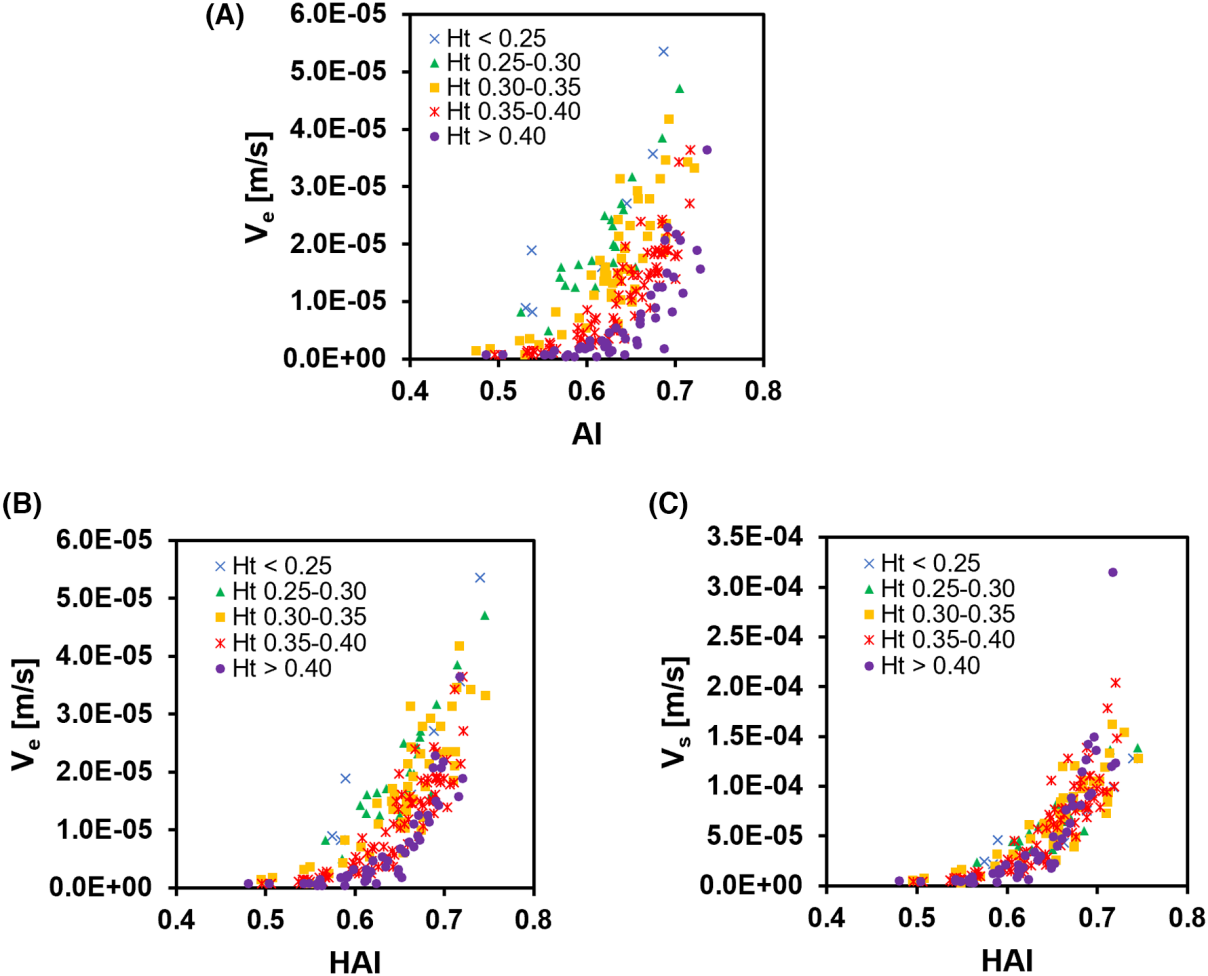
Relationship between the aggregation index and sedimentation velocities. (A) Relationship between the AI measured over a 5‐s interval and erythrocyte sedimentation. (B) Relationship between the HAI measured over a 5‐s interval and erythrocyte sedimentation velocity. (C) Relationship between the HAI measured over a 5‐s interval and the sedimentation velocity of an erythrocyte aggregate divided by the hindered settling effect.

Figure 2A shows the relationship of the sedimentation velocity *V*_e_ with AI. *V*_e_ at each Ht range (<0.25, 0.25–0.30, 0.30–0.35, 0.35–0.40, and >0.40) increased exponentially with AI, with a higher Ht increasing AI and decreasing *V*_e_ (overall Ht range, 0.214–0.523). Before Ht correction of AI, the correlation between *V*_e_ and AI was not strong (*r* = 0.645, *p* < 0.001) due to the Ht difference. In contrast, the relationship after Ht correction showed a narrowed distribution in the horizontal direction, as shown in Figure 2B. Furthermore, as shown in Figure 2C, the relationship between HAI and the sedimentation velocity of a single erythrocyte *V*_s_, obtained by dividing *V*_e_ by the hindered settling coefficient, exhibited a narrowed distribution in the vertical direction. These results agreed with the previously reported findings obtained with fibrinogen‐added healthy blood.^18^

### 3.2 Comparison of ESR calculated with HAI and Ht to WG ESR

**FIGURE 3:**
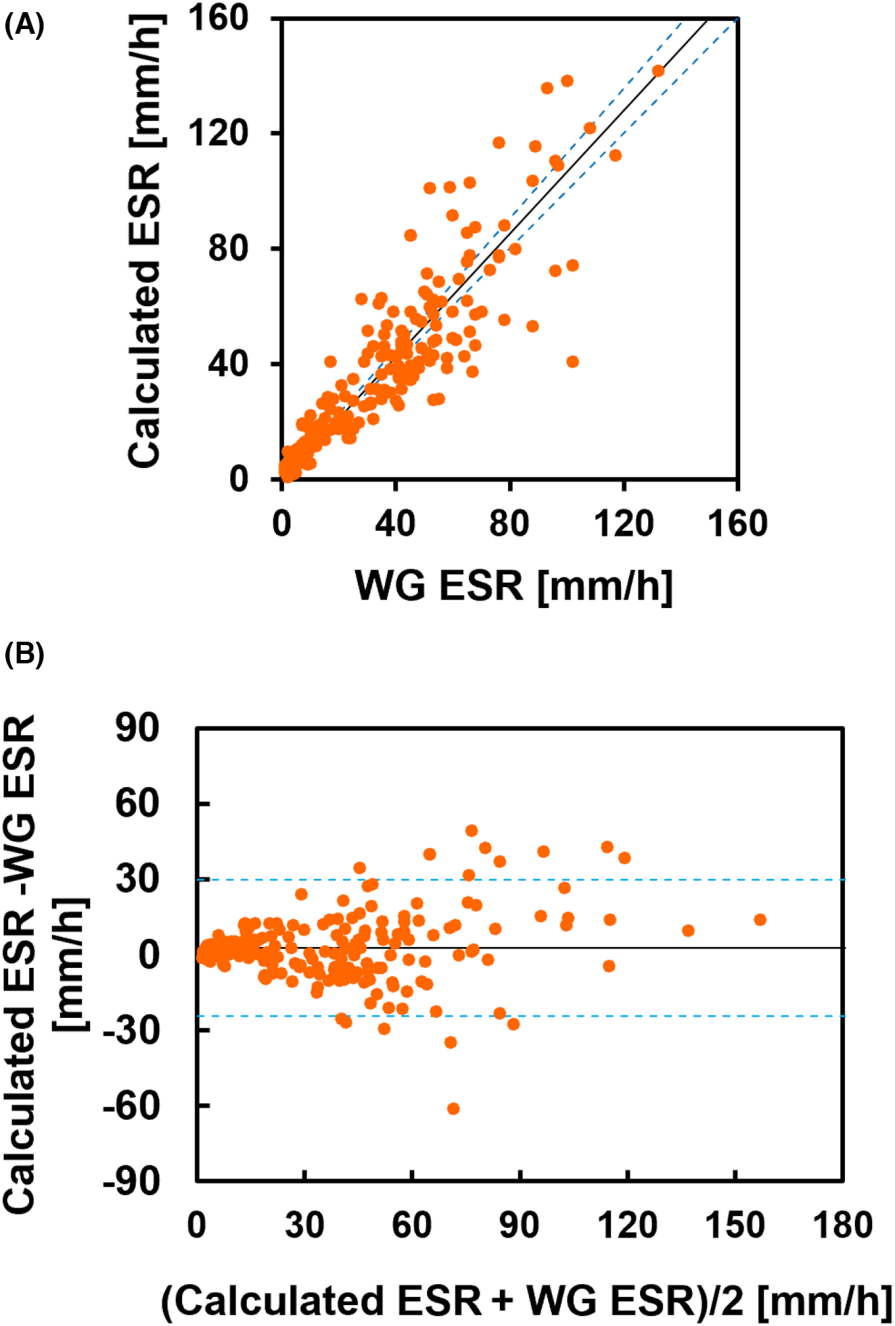
Comparison of the ESR calculated using HAI and Ht with WG ESR. (A) Scatter plot of the calculated ESR for WG ESR. Correlation coefficient, *r* = 0.899 (*p* < 0.001). The regression line calculated by the Passing–Bablok method is shown as a solid line, and the regression lines at the upper and lower limits of the 95% confidence interval are shown as dotted lines. (B) Bland–Altman plot for the calculated ESR and WG ESR. The solid line represents the bias (2.76). The dotted line indicates 1.96 SD.

**FIGURE 4:**
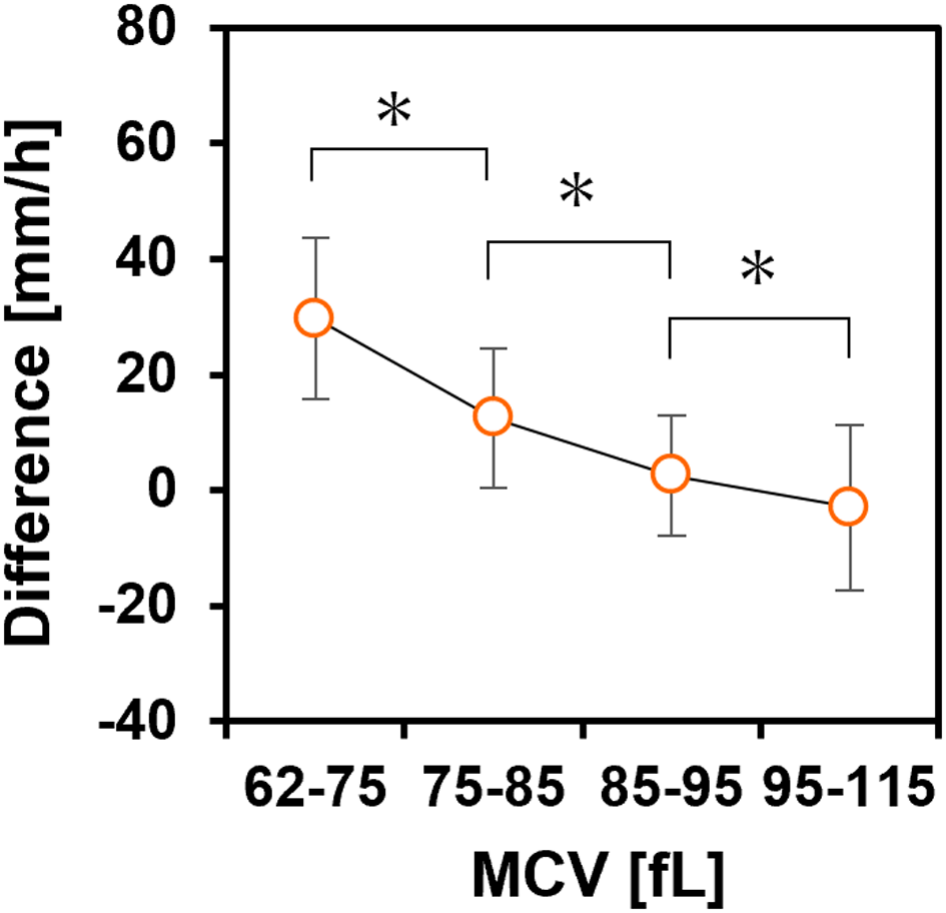
Mean difference in the ESR calculated using HAI and Ht from WG ESR for each MCV range. Error bars indicate SD. *p < 0.05 (Student's *t*‐test).

Figure 3A shows a comparison of the ESR value calculated using HAI and Ht with that of the Westergren method. The correlation between the ESR obtained by our method and WG ESR was high (*r* = 0.899, *p* < 0.001). The slope of the linear regression equation was 1.068 (95% confidence interval, 0.998–1.127), and the intercept was 0.559 (95% confidence interval, −0.433 to 1.538). Figure 3B shows the Bland–Altman plots: the difference was relatively small for an ESR below 20 mm/h but tended to increase in both positive and negative directions from 30 to 120 mm/h. Figure 4 shows the mean difference of the calculated ESR to WG ESR for each of the four MCV ranges (62–75 fL [*n* = 8], 75–85 fL [*n* = 18], 85–95 fL [*n* = 109], and 95–115 fL [*n* = 68]). The differences became larger as MCV increased, indicating that correction for the effect of Ht on the AI alone was insufficient to correct for the effect of the individual differences in MCV.

### 3.3 Validation of MCV correction

**FIGURE 5:**
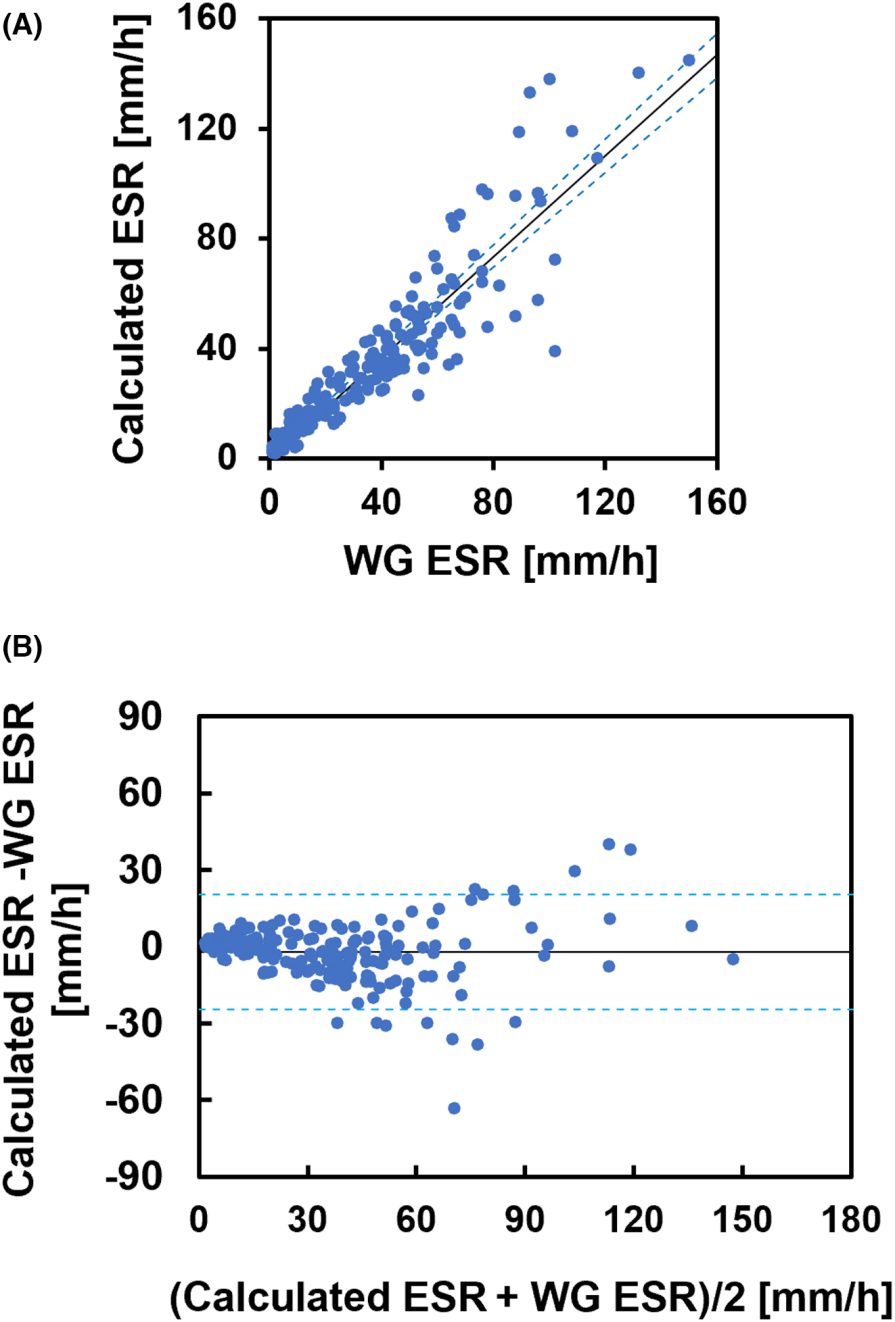
Comparison of the ESR calculated using HAI, Ht, and MCV with WG ESR. (A) Scatter plot of the calculated ESR for WG ESR. Correlation coefficient, *r* = 0.920 (*p* < 0.001). The regression line calculated by the Passing–Bablok method is shown as a solid line, and the regression lines at the upper and lower limits of the 95% confidence interval are shown as dotted lines. (B) Bland–Altman plot for the calculated ESR and WG ESR. The solid line represents the bias (−2.17). The dotted line indicates 1.96 SD.

**FIGURE 6:**
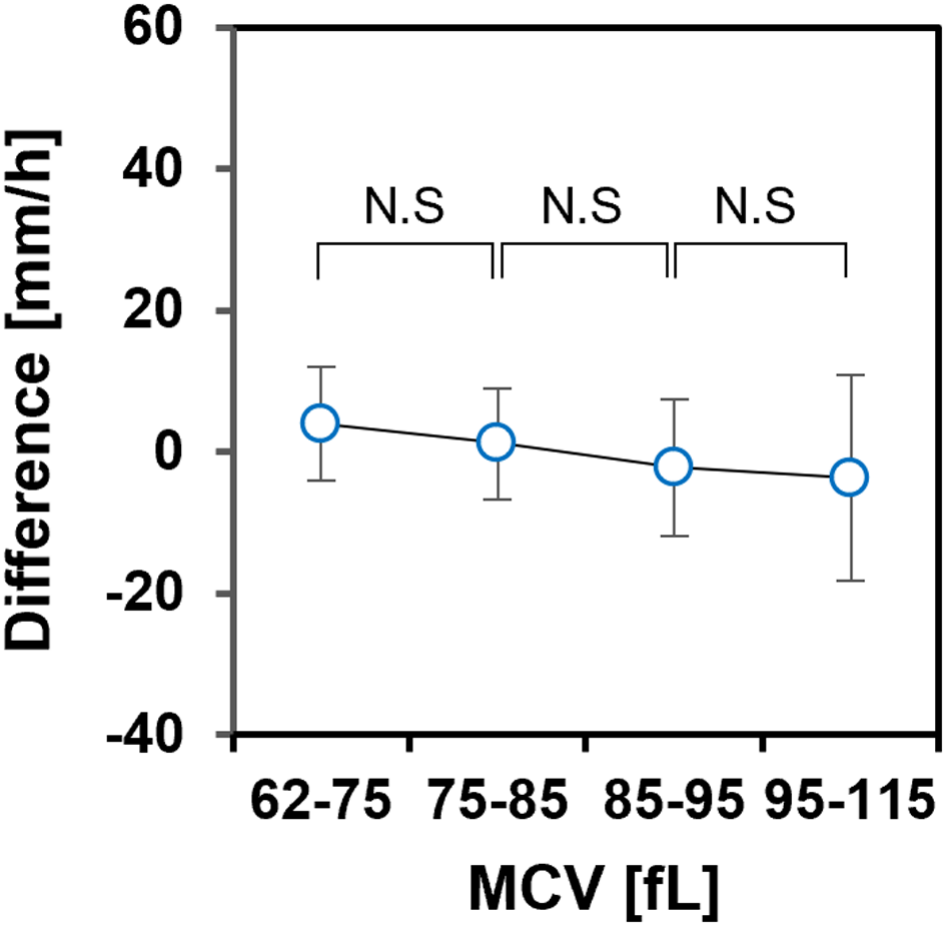
Mean difference in the ESR corrected for MCV from WG ESR for each MCV range. Error bars indicate SD. N.S: Not significant.

Figure 5A compares the ESR calculated with the effective diameter of erythrocytes from individual MCVs and WG ESR. Calculations with MCV improved the correlation coefficient between the calculated values and WG ESR (*r* = 0.920, *p* < 0.001). In the Bland–Altman plot shown in Figure 5B, the difference was reduced at ESRs below 90 mm compared to the case without MCV correction. As shown in Figure 6, there was no difference in the mean differences for each MCV range, indicating that the MCV dependence was effectively reduced by the correction for MCV.

## 4 DISCUSSION

In this study, we demonstrated that the ESR calculated using HAI, the AI corrected for the influence of Ht, correlated well with the value obtained by the Westergren method in clinical specimens. Similar to previous work,^6^ a comparison of ESR and AI without Ht correction showed a poor correlation, which was obviously due to Ht (Figure 2A). We showed that Ht correction of AI and consideration of the influence of the hindered settling occurring in concentrated blood cells effectively improved the ESR calculation in a variety of clinical specimens (Figure 2B,C). We then confirmed in clinical specimens that the sedimentation velocity of a single erythrocyte could be calculated as an exponential function of HAI. These results demonstrate that the values obtained by our erythrocyte aggregation measurement conform to Stokes' law, which has been widely used to explain the principle of ESR.^19^, ^20^, ^30^ Therefore, the values obtained by this method are similar to those of the Westergren method. Furthermore, our method provides ESR values similar to those of the Westergren method regardless of Ht, in contrast to alternative methods that show deviations from the values of the Westergren method due to Ht.^13^, ^14^ Because the ICSH recommends that attention be paid to differences in the methods,^15^ understanding their detailed principles and formulae is helpful for accurately interpreting the test values.

It had previously been difficult to evaluate the MCV dependence of the differences between calculated and measured values because Ht has a much greater effect on erythrocyte aggregation parameters than MCV. We succeeded in evaluating the MCV dependence of the differences by using Ht correction of an aggregation parameter. However, for some samples in the high ESR range of 100–120 mm, which is the transient phase in which the sedimentation rate decelerates from a constant velocity, the calculated ESR values were larger than those of WG ESR by more than 30 mm (Figure 5B). We assumed that the coefficient of Mayer's empirical equation,^30^ which determines the transient time, was not optimal for our conditions. This deviation is also considered to be caused by plasma proteins, the erythrocyte membrane charge, and erythrocyte deformability.^31^, ^32^, ^33^ In particular, a fixed plasma viscosity value was used in this method, but this value varies among specimens. Some samples with significantly lower calculated values than WG ESR tended to show lower concentrations of total protein, indicating that the lower calculated values may be related to the overestimated plasma viscosity. We have not discussed the slope and intercept for the Westergren method because the optimal coefficients of the equation were determined by data fitting. However, the range of their 95% confidence interval was sufficiently small, indicating that our proposed method is superior. In addition, our method can alternatively use another aggregation parameter, namely the aggregation half‐time (*t*_1/2_), because there is an inverse relationship between AI and *t*_1/2,_ ^34^, ^35^ which we confirmed under our conditions.

This study demonstrates that our rapid and accurate ESR calculation method and use of MCV correction are valid for clinical specimens. However, there were some limitations in the statistical analysis. All variables except for Ht were non‐normal, as shown in Table S1 and Figure S1, and in such cases, a non‐parametric method such as Spearman's correlation coefficient is appropriate. The values of Spearman's correlation coefficient were higher than those of Pearson's correlation coefficient (Table S2), but this was because Spearman's correlation coefficient is not affected by outliers or monotonic changes, which can be a problem in a clinical laboratory. Therefore, we believe that the use of Pearson's correlation coefficient, as in previous reports,^11^, ^13^ is appropriate for the accurate comparative evaluation of clinical instruments. Another limitation is that the sample size selection and clinical performance evaluation recommended by the ICSH^15^ were not fully performed.

In conclusion, we successfully demonstrated that the simultaneous measurement of CBC, including Ht and MCV measurements, and erythrocyte aggregation in as little as 5 s provides a better correlation with WG ESR than previously reported rapid ESR assays.^13^, ^14^ In hematology tests, where ESR and CBC are frequently ordered simultaneously, our method, which can obtain ESR and CBC within 3 min with a single aspiration of only an 80 μL EDTA blood sample, will greatly contribute to improving the efficiency of hematology tests and diagnosis.

## AUTHOR CONTRIBUTIONS

M.H. designed the study, performed data analysis, and wrote the manuscript. Both authors reviewed and revised the manuscript.

## CONFLICT OF INTEREST STATEMENT

Makoto Higuchi is a current employee of the Nihon Kohden Corporation. The results of this research are patent pending, with Makoto Higuchi as the inventor and Nihon Kohden Corporation as the applicant.

## Supporting information


Figure S1

## Data Availability

The analyzed datasets generated during the study are available from the corresponding author upon reasonable request.
