# Supplementary material for: Determination of the erythrocyte sedimentation rate using the hematocrit‐corrected aggregation index and mean corpuscular volume
Source: J Clin Lab Anal. 2023 Apr 1;37(6):e24877. doi: 10.1002/jcla.24877 (PMC10156103; doi:10.1002/jcla.24877)
Supplement: Supplementary file 1 — Figure S1 [file JCLA-37-e24877-s001.docx]

Table S1 Statistical data and normality analysis of the variables.

|  | n | Min | Max | Mean | SD | Normality Shapiro-Wilk test | *p-*value |
| --- | --- | --- | --- | --- | --- | --- | --- |
| WG ESR [mm/h] | 203 | 1 | 150 | 36.1 | 28.7 | Non-normal | <0.0001 |
| Calculated ESR without MCV correction [mm/h] | 203 | 0.9 | 164.0 | 38.8 | 31.7 | Non-normal | <0.0001 |
| Calculated ESR with MCV correction [mm/h] | 203 | 1.9 | 145.0 | 34.0 | 28.5 | Non-normal | <0.0001 |
| Ht [%] | 203 | 21.4 | 52.3 | 36.3 | 5.7 | Normal | 0.14 |
| MCV [fL] | 203 | 62.7 | 114.1 | 92.0 | 7.8 | Non-normal | <0.0001 |
| AI | 203 | 0.47 | 0.74 | 0.63 | 0.05 | Non-normal | <0.001 |
| HAI | 203 | 0.48 | 0.75 | 0.64 | 0.06 | Non-normal | <0.0001 |

The Shapiro–Wilk test was performed using XLSTAT (Addinsoft, New York, NY).

Table S2 Correlation coefficients between the WG ESR and the calculated ESR using the Pearson’s and Spearman’s correlation coefficients.

|  | Pearson’s correlation coefficient *r* | Spearman’s correlation coefficient ρ |
| --- | --- | --- |
| Westergren ESR and calculated ESR (without MCV correction) | 0.899 | 0.922 |
| Westergren ESR and calculated ESR (with MCV correction) | 0.920 | 0.953 |

Calculation of the Pearson’s and Spearman’s correlation coefficients were performed using XLSTAT (Addinsoft, New York, NY).


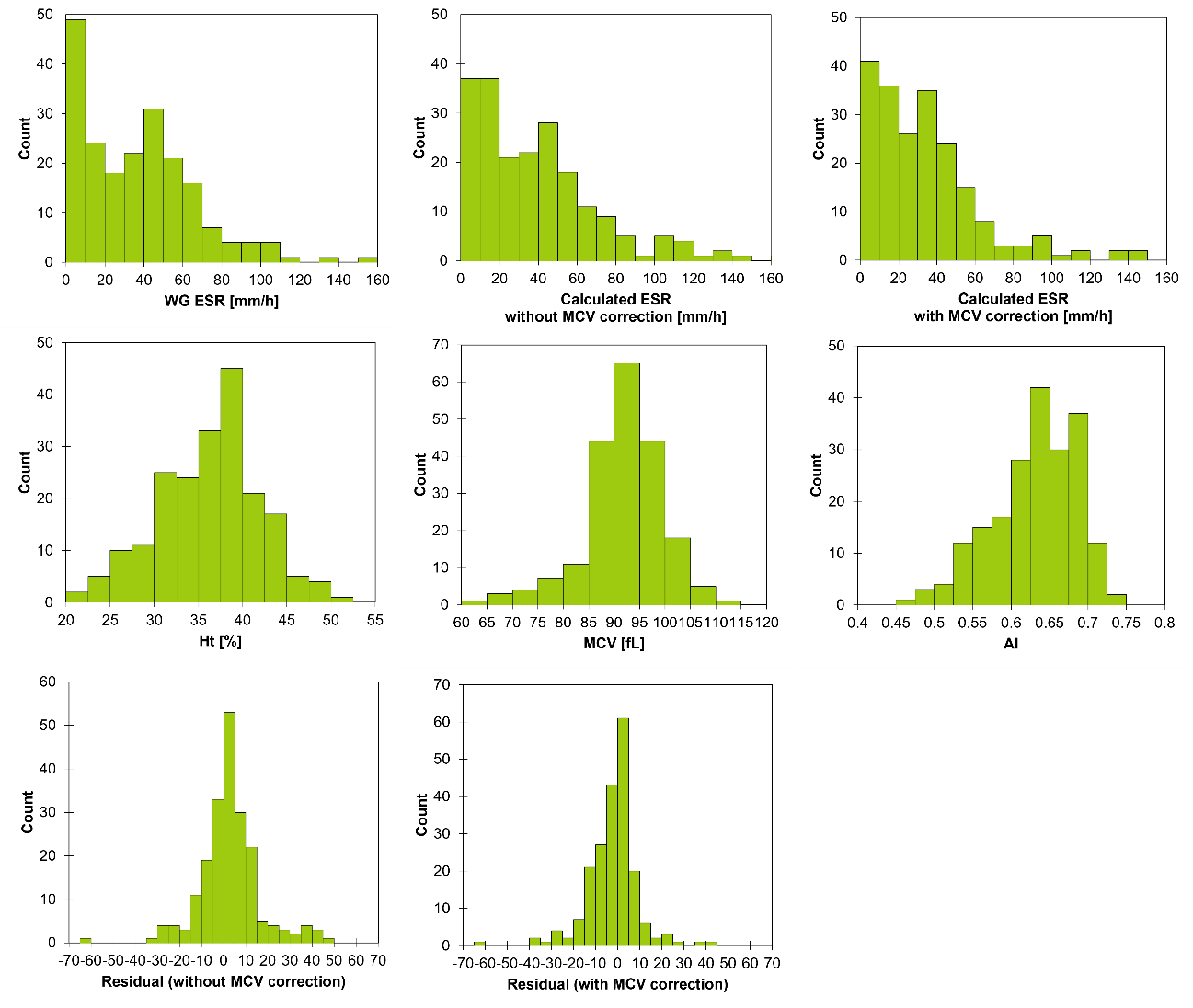


Fig. S1 Histograms of the variables and the residuals.

WG ESR: ESR value obtained by the Westergren method.

Residual = Calculated ESR − WG ESR.
